# Chaos in Optomechanical Systems Coupled to a Non-Markovian Environment

**DOI:** 10.3390/e26090742

**Published:** 2024-08-30

**Authors:** Pengju Chen, Nan Yang, Austen Couvertier, Quanzhen Ding, Rupak Chatterjee, Ting Yu

**Affiliations:** 1 Center for Quantum Science and Engineering, Department of Physics, Stevens Institute of Technology, Hoboken, NJ 07030, USA; pchen9@stevens.edu (P.C.); nyang.hust@gmail.com (N.Y.); acouvert@stevens.edu (A.C.); qding3@stevens.edu (Q.D.); 2 Deptartment of Applied Physics, New York University Tandon School of Engineering, Brooklyn, NY 11201, USA; rc5479@nyu.edu

**Keywords:** chaos, non-Markovian environment

## Abstract

We study the chaotic motion of a semi-classical optomechanical system coupled to a non-Markovian environment with a finite correlation time. By studying the emergence of chaos using the Lyapunov exponent with the changing non-Markovian parameter, we show that the non-Markovian environment can significantly enhance chaos. It is observed that a non-Markovian environment characterized by the Ornstein–Uhlenbeck type noise can modify the generation of chaos with different environmental memory times. As a comparison, the crossover properties from Markov to non-Markovian regimes are also discussed. Our findings indicate that the quantum memory effects on the onset of chaos may become a useful property to be investigated in quantum manipulations and control.

## 1. Introduction

Optomechanics studies the interaction between light and mechanical systems [1,2]. The nonlinear optical and mechanical interactions have provided a new platform to realize quantum information processing [3,4] and quantum sensing [5], and to test the fundamental quantum physics such as decoherence [6], quantum entanglement [7,8], classical dynamical gauge fields [9], and Parity-Time (PT) symmetry breaking [10], to name a few. In addition, many novel features of optomechanical systems involving chaotic properties have been investigated in various interesting settings [1,2,3,4,5,6,7,9,10,11,12,13,14,15,16,17], one of the interesting physical problems in employing optomechanical systems is the chaotic motion in an optomechanical system coupled to a dissipative environment [18]. Such study has opened up new opportunities in studying the onset of chaos dynamics has have emerged from the non-linearity of the optomechanical systems in a semi-classical regime, and for a Markov environment, it has shown several interesting features on how the system parameters such as the detuning, the optical and mechanical oscillator coupling and the external pumping power can affect the chaotic motion. This optomechanical platform has shed new light on the study of chaotic motion induced by quantum systems, which has attracted a wide spectrum of interests over recent decades [19,20,21,22,23,24,25,26,27].

The sensitivity of chaotic motion to the parameters of the optomechanical system raises an interesting question: are there any other physical parameters that can play an important role in altering the regular and chaotic motion of an open optomechanical system? One direct answer to this question is to go beyond the assumption of Markov environment. The purpose of this paper is to study the regular and chaotic motion of nonlinear optomechanical systems coupled to a non-Markovian environment [28,29,30]. It is observed that a non-Markovian environment characterized by an Ornstein–Uhlenbeck type noise can modify chaotic motion with different environmental memory times. The advantages of choosing an Ornstein–Uhlenbeck type noise is that the environmental memory times may be conveniently characterized by a single parameter γ that will provide a useful model to study the crossover properties from non-Markovian to Markov regimes (γ→∞). Moreover, our model has shown that the environmental memory time defined in this model may become a useful property to be investigated in quantum manipulations and control [31,32]. More specifically, we will study the dynamics of the optomechanical system coupled to a non-Markovian environment and explore the emergence of chaotic motion by using a Lyapunov exponent with varied environmental memory times [33,34,35,36,37,38,39,40,41]. As a result, our non-Markovian approach also provides a convenient pathway to the well-known Markov limit [42,43,44,45,46].

This paper is organized as follows: in Section II, we consider combined mechanical and optical modes coupled to two heat baths. By employing the approximate non-Markovian master equation, one can effectively simulate the dynamics of the optomechanical system. In Section III, we study the chaotic dynamics of the optomechanical system embedded in a non-Markovian environment. These results are used to illustrate how the environmental memory affects the onset of chaos in various parameter settings. As a comparison, the crossover properties from Markovian to non-Markovian regimes are also discussed. In Section IV, we make some comments on several physical issues that are relevant to the discussions presented in this paper and further extension of the present study. Technical details are relegated to the Appendix A.

## 2. Optomechanical Model Coupled to a Non-Markovian Environment

**Figure 1:**
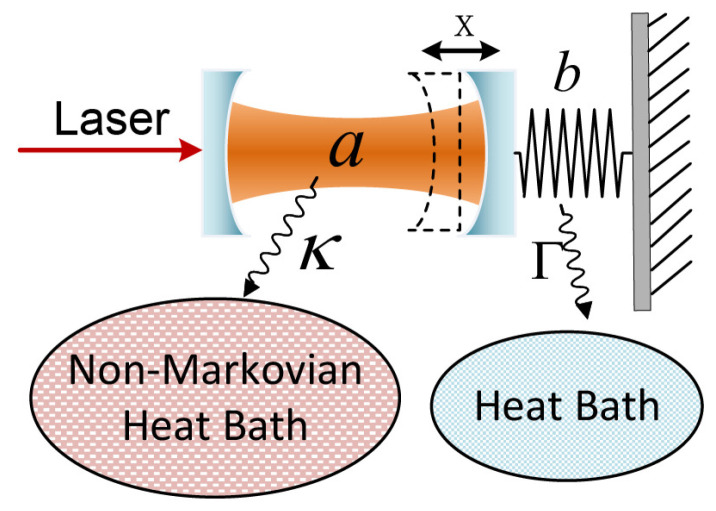
Schematic diagram of the optomechanical system, where the light field is considered to be in a non-Markovian environment. The mechanical bath is set to be Markov to account for mechanical loss.

In this section, we consider an optomechanical system coupled to a non-Markovian environment. We will use this model to investigate the interrelationship between the chaos and the environmental memory. The optomechanical system consists of a mechanical mode coupled with an optical mode, as exhibited in Figure 1.

The optomechanical system under consideration is described by the Hamiltonian [1] (setting ℏ=1)

(1) $$H_{s}=\left[-\Delta_{\omega }+g_{0}\left(b+b^{†}\right)\right]a^{†}a+\Omega b^{†}b+\alpha_{L}\left(a^{†}+a\right),$$

where *a*$\left(a^{†}\right)$ and *b*$\left(b^{†}\right)$ are the annihilation (creation) operators of the optical mode and mechanical mode, which satisfy the commutation relations $[a,a^{†}]=1$ and $[b,b^{†}]=1$, respectively. Here, Ω is the mechanical cantilever frequency, and $\Delta_{\omega }$ is the detuning parameter, which is defined as $\Delta_{\omega }\equiv \omega_{L}-\omega_{\mathrm{cav}}$, where $\omega_{L}$ is the pumping laser frequency and $\omega_{\mathrm{cav}}$ is the resonant frequency of the cavity mode *a*. $\alpha_{L}$ is the pumping laser amplitude and $g_{0}$ is the coupling constant between the two mechanical and optical modes.

Since the optomechanical system is modeled as an open system, the cavity has radiative loss and the cantilever has mechanical damping with respect to their local environments. The two baths are assumed to be Bosonic, with the optical bath set to be non-Markovian, which is the main focus of our study and may be adjusted experimentally [47]. The mechanical bath is considered Markov to account for mechanical loss. The damping parameters of the optical and mechanical baths are, respectively, κ/Ω=1 and $\Gamma /\Omega =10^{-3}$. Here, the mutual correlation between two environments is omitted as their interaction strength is much weaker than that between the systems and environments.

Using the first-order, so-called post-Markov approximation of the non-Markovian quantum state diffusion (NMQSD) equation [34], the master equation takes the following form (details can be seen in Appendix A)

(2) $$\dot{\rho }=-i\left[H_{s},\rho \right]+\Gamma D\left[b,\rho \right]+\left\{f_{0}\left(t\right)\left[a\rho ,a^{†}\right]+if_{1}\left(t\right)\left[a^{†},\left[H_{s},a\right]\rho \right]+f_{2}\left(t\right)\left[a^{†},\left[a^{†},a\right]a\rho \right]+H.C.\right\},$$

where H.C. stands for Hermitian conjugate. The so-called Lindblad term $D\left[b,\rho \right]=\left\{b\rho b^{†}-\frac{1}{2}\left(b^{†}b\rho +\rho b^{†}b\right)\right\}$ represents the effect of the Markov mechanical bath. And the non-Markovian environment effects represented by the time-dependent coefficients $f_{0}$, $f_{1}$, $f_{2}$ are given by

(3) $$\begin{array}{cc} \; & f_{0}\left(t\right)=\int_{0}^{t}\alpha \left(t,s\right)\;ds,\\ \; & f_{1}\left(t\right)=\int_{0}^{t}\alpha \left(t,s\right)\left(t-s\right)\;ds,\\ \; & f_{2}\left(t\right)=\int_{0}^{t}\int_{0}^{s}\alpha \left(t,s\right)\alpha \left(s,u\right)\left(t-s\right)\;du\;ds. \end{array}$$

where α(t,s) is the Ornstein–Uhlenbeck (O-U) type correlation function,

(4) $$\alpha \left(t,s\right)=\frac{\kappa \gamma }{2}e^{-\gamma |t-s|},$$

where $\tau_{\mathrm{env}}=1/\gamma$ is the environmental memory time, and κ is the optical damping rate where κ/Ω=1.

For convenience, we express the system parameters in units of Ω and introduce the dimensionless time parameter τ=Ωt. Additionally, we introduce a dimensionless parameter [18,48,49]

(5) $$P=\frac{8\alpha_{L}^{2}g_{0}^{2}}{\Omega^{4}}.$$

The pumping parameter *P* represents the strength of the laser input of the cavity.

We use the re-scaled mean values of the creation and annihilation operators $\alpha_{1}=\left[\Omega /\left(2\alpha_{L}\right)\right]\langle a\rangle$, $\beta_{1}=\left(g_{0}/\Omega \right)\langle b\rangle$ to represent photon and phonon modes. In the bad-cavity limit, namely, where the leakage rate κ of the cavity is sufficiently high, such that $g_{0}/\kappa \ll 1$, the semi-classical (SC) equations of motion are given by (the equations governing $\alpha_{1}^{*}$ and $\beta_{1}^{*}$ may be obtained easily),

(6) $$\begin{array}{cc} \frac{d\alpha_{1}}{d\tau }= & -i\left(1+f_{1}\right)\left\{\left(\beta_{1}+\beta_{1}^{*}\right)\alpha_{1}-\frac{\Delta_{\omega }}{\Omega }\alpha_{1}+\frac{1}{2}\right\}-\frac{f_{0}+f_{2}}{\Omega }\alpha_{1},\\ \frac{d\beta_{1}}{d\tau }= & -i\left\{\frac{P}{2}{\left|\alpha_{1}\right|}^{2}+\beta_{1}\right\}-\frac{\Gamma }{2\Omega }\beta_{1}, \end{array}$$

where we used the SC approximation $\langle \left(b^{†}+b\right)a\rangle \approx \langle b^{†}\rangle \langle a\rangle +\langle b\rangle \langle a\rangle$, which ignores all photon–phonon correlations. The coefficients $f_{0}$, $f_{1}$ and $f_{2}$ are time-dependent and encode the information about the non-Markovian environment.

## 3. Chaos in Non-Markovian Environments

Effects of the non-Markovian environment on the chaotic motion of the optomechanical system is the focus of this section. The optomechanical systems have proved their sensitivity to parameter changes of the input power *P* and the detuning $\Delta_{\omega }$ in a Markov environment [18]. The new elements brought into the nonlinear equations of motion (6) are the time-dependent coefficients, one would expect that the chaotic motions should be modified by introducing more variable parameters. The simulation methods used below allow us to explore how the non-Markovian memory time influences the chaos generation in the optomechanical system.

Our simulations are mainly based on observing the optomechanical system while changing the memory time of the optical bath represented by the parameter γ (the inverse of memory time). Furthermore, we vary the detuning $\Delta_{\omega }$ and the pumping strength *P* to obtain a comprehensive picture of the chaos distribution of our system. The initial states are set to be the vacuum states. We use the maximal Lyapunov exponent (LE) as the indicator of chaos, which is calculated using Wolf’s method of phase reconstruction [50] at the long-term limit and excludes the initial transient stage. The positive LE implies the onset of chaos.

### 3.1. Simulation Results

First, the phase space description provides a powerful tool to study chaos in Markov and non-Markovian environments. By taking the real and imaginary parts of the time series of $\alpha_{1}$ and $\beta_{1}$ from Equation (6), we can generate the four-dimensional phase diagrams for different memory times (the inverse of γ).

**Figure 2:**
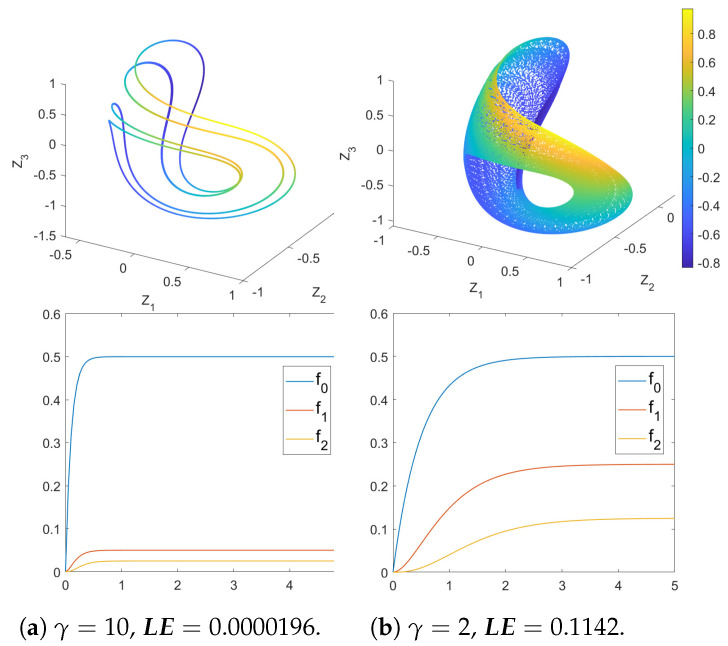
The top 2 figures are phase diagrams with different memory times at $\Delta_{\omega }=-0.65$, P=1.37; such a point is chosen based on the bifurcation diagrams (Figure 3). The coordinates $\left(Z_{1},Z_{2},Z_{3},Z_{4}\right)$ are the real and imaginary parts of $\alpha_{1}$ and $\beta_{1}$. The 4th coordinate $Z_{4}$ is represented by scaled colors. As memory time increases (γ decreases), the dynamics go from regular to chaotic, as indicated by both the phase diagrams and LE. The bottom 2 figures show the evolution of $f_{0}\left(t\right)$, $f_{1}\left(t\right)$ and $f_{2}\left(t\right)$ with the integration time τ=5. The decrease of γ not only slows down the convergence of $f_{i}$ functions, it also changes the stable values of $f_{1}$ and $f_{2}$.

**Figure 3:**
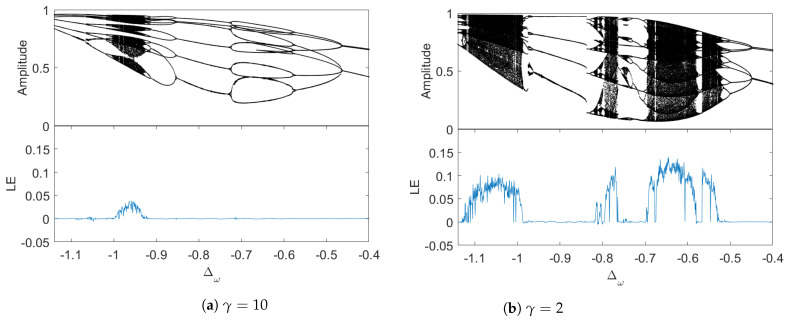
Bifurcation diagrams and the corresponding maximum Lyapunov exponents (LE) with different memory times at P=1.37. In (**a**), the chaotic region is bounded within a small segment $\Delta_{\omega }\in \left[-1.03,-0.92\right]$. In (**b**), one could see more bifurcation, the chaotic region expands to $\Delta_{\omega }\in \left[-1.13,-0.99\right]$, and a new chaotic region $\Delta_{\omega }\in \left[-0.83,-0.52\right]$ emerges, with some inter-adjacent regular regions inside.

Figure 2 illustrates the system dynamics under different memory times for a chosen point (P=1.37 and $\Delta_{\omega }=-0.65$), such a point is chosen based on the bifurcation diagrams (Figure 3), where a non-chaotic point when γ=10 (LE=0.0000196) becomes chaotic when γ=2 (LE=0.1142), as indicated by both phase diagrams and LE. It is observed that the increase in memory time (decrease in γ) results in the system changing from regular to chaotic, such a transition is caused by the change in evolutions of $f_{0}\left(t\right)$, $f_{1}\left(t\right)$ and $f_{2}\left(t\right)$ (simply denoted as $f_{i}$), as is shown in Figure 2. Note that we take the integration time of $f_{i}$ for just τ=5 since they converge very fast, and the integration time for the phase diagrams is τ=10000 to accommodate for chaos. The increase in memory time slows down the convergence of $f_{i}$ functions while changing the stable values of $f_{1}$ and $f_{2}$.

Next, we plot the bifurcation diagrams of the cantilever oscillation. Fixing P=1.37, at γ=10, the period-doubling bifurcation takes place, as is exhibited in Figure 3a, where the chaotic region is bounded within a small segment $\Delta_{\omega }\in \left[-1.03,-0.92\right]$. With the same *P*, we change γ to 2. Figure 3b shows more bifurcations, and the chaotic region expanding to $\Delta_{\omega }\in \left[-1.13,-0.99\right]$ while a new chaotic region at $\Delta_{\omega }\in \left[-0.83,-0.52\right]$ emerges, with some inter-adjacent regular regions inside. The comparison between Figure 3a,b indicates that the longer memory time expands chaotic regions. Physically, this is a very interesting observation. It indicates that an engineered environment with finite correlation times may be used to control the onset of chaos and system dynamics. Such an idea may be of interest in quantum measurement and control. Note that γ=2 is not far away from the Markov limit, but it still causes significant changes to chaotic dynamics, further indicating that the chaos is very sensitive to parameter changes, including the memory time.

**Figure 4:**
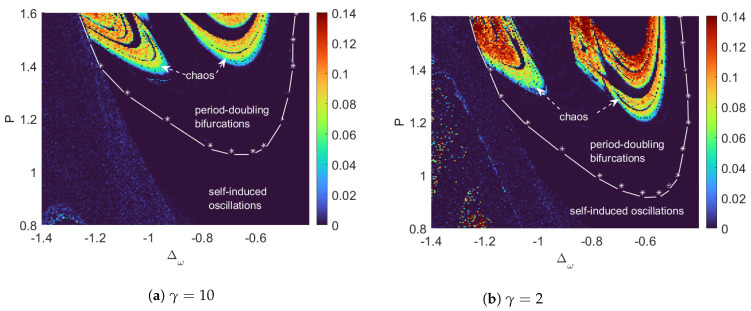
Pictures of chaotic regions of the optomechanical systems with different memory times plotted in the *P*-$\Delta_{\omega }$ plane. The color scale shows the value of the maximal Lyapunov exponent (LE) of every data point. White stars represent data points extracted from bifurcation diagrams, while dashed lines interpolate between the data. Comparing (**a**,**b**), the chaotic area expands as the memory time becomes longer (γ decreases).

To summarize the appearances of chaos, the LE of every data point is plotted to form global chaos landscapes, as is shown in Figure 4a for γ=10 and Figure 4b for γ=2. The parameter ranges are set to be P∈[0.8,1.6] and $\Delta_{\omega }\in \left[-1.4,-0.4\right]$, which are consistent with the current technology and contain interesting chaotic behaviors [18]. As the pumping strength increases, we see more chaotic motion [18,48,49]. In both cases, the chaotic regions have shown some fine structures of inter-adjacent regular regions. For our model, we have shown that the non-Markovian environment may expand the chaotic regions while lowering the pumping power threshold for chaos generation.

**Figure 5:**
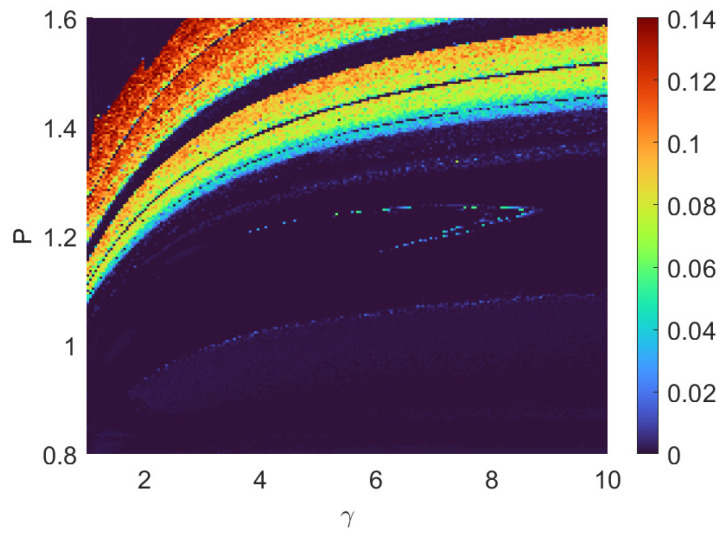
Fixing $\Delta_{\omega }=-0.6$, the plot shows the chaos landscape of γ vs. *P*. The pumping power threshold for chaos generation decreases as memory time increases (γ decreases). Also, each γ corresponds to a specific vertical line of chaos distribution, which could be exploited as a tool of measuring an unknown material’s memory time.

The chaos landscape based on γ vs *P* can be seen in Figure 5, with $\Delta_{\omega }=-0.6$. The pumping power threshold for chaos generation decreases as memory time increases (γ decreases). Each γ corresponds to a specific vertical line of chaos distribution, due to the chaos’ sensitivity to parameters. The uniqueness of the chaos distribution regarding each specific γ could be the tool of ultra-sensitive measurement of memory time of an unknown material.

We have seen that the increase in memory time can expand chaotic areas in the parameter plane and decrease the pumping threshold for chaos generation. The non-Markovian environment has modified the dissipative rate (time-dependent), and as such, it has modified the dynamics of chaotic motion. Subsequently, the non-Markovian properties of the environment have brought about several new features such as lowering the pumping threshold while expanding chaotic regions across the map. It is noted that a similar phenomenon was also observed in that the environment memory may enhance entanglement generation in a non-Markovian regime [51].

### 3.2. The Comparison between Markov and Non-Markovian Regimes

How is the chaotic motion in the optomechanical system coupled to a non-Markovian environment? There is no single prescription for the chaos behaviors because the onset of chaos is sensitive to the different physical parameters such as the detuning, pumping power and the environmental memory time. We now turn to the comparison of the Markov and non-Markovian regimes. We find the post-Markov approximation particularly convenient for this purpose as the post-Markov regime provides the first-order correction to the well-known Markov regime.

**Figure 6:**
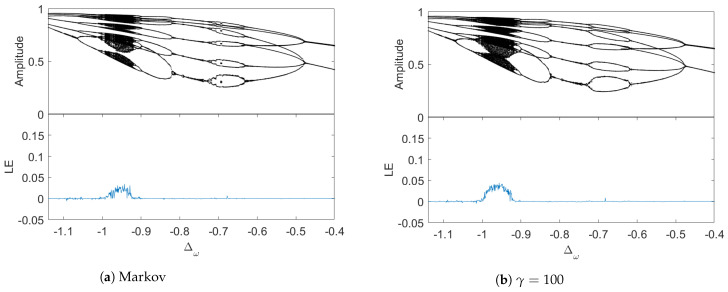
Bifurcation diagrams at P=1.4. They show the transition of the environment from a Markovian regime to non-Markovian.

We begin by choosing the Markov limit (i.e., γ→∞), and the correction function is described by a delta function: α(t−s)=κδ(t−s), then $f_{0}=\kappa /2$, $f_{1}=0$ and $f_{2}=0$, substitution of these constant coefficients into Equation (6) and setting P=1.4, we can numerically generate the bifurcation diagram and the corresponding LE of the Markov case (Figure 6a). As a comparison, we also plotted the same bifurcation diagram by using the non-Makovian equations with the parameter γ=100 (Figure 6b). We note, however, that, in the long-time limit (t→∞), the coefficients with γ=100 are $f_{0}=0.5$, $f_{1}=0.005$ and $f_{2}=0.0025$; though $f_{1},f_{2}$ are small, they are not zeros (as in the case of Markov approximation). Our numerical simulations show the sensitivity of chaotic motion in the presence of non-zero coefficients $f_{1},f_{2}$. Clearly, as γ becomes larger and larger, the distinction in the bifurcation diagrams will be smaller and smaller, approaching the Markov limit (Figure 6a). We have numerically demonstrated that, as the environmental memory time $\tau_{\mathrm{env}}=1/\gamma$ decreases, the chaotic regions will shrink and converge to the Markov case.

## 4. Conclusions

The chaotic motion that emerges from open quantum systems is important for a better understanding of quantum open system dynamics, and it is also of great interest for many physical applications such as quantum measurement and quantum sensing. Based on the optomechanical system coupled to a non-Markovian environment describing the finite correlation time $\tau_{\mathrm{env}}=1/\gamma$, we have shown that the finite environmental memory times can affect chaos generation in various interesting parameter ranges. It is seen that the environmental memory effect effectively enlarges the chaotic region while lowering the pumping power threshold for chaos generation. Such a phenomenon provides an additional parameter to control chaotic motion in optomechanical systems [52,53]. There are many interesting questions remaining, such as the relationship between optomechanical entanglement generation and the chaos, the high-order non-Markovian effects on the chaos behaviors, and, given that the non-Markovian process is time correlated, its relationship with the dynamics of out-of-time-ordered correlators (OTOCs) [54,55,56]. As the signature of quantum chaos could be extremely relevant in our study, these will be left for future publications.

## Data Availability

Data underlying the results presented in this paper are public and can be found in this GitHub repository: https://github.com/DOGECHANN/Chaos-in-Non-Markovian-Optomechanical-Systems (accessed on 1 October 2022).

## Appendix A. The Non-Markovian Quantum State Diffusion (NMQSD) and Master Equation

For the convenience of the general readership, the following section provides details about the master equation used in this paper.

Both environments are set to be at zero temperature and separate from each other. Since the Markov environment is well-understood and its formation can be added to the master equation easily, we can focus on the optical bath and use the non-Markovian quantum state diffusion (QSD) equation to derive the master equation.

The Bosonic bath for the optical mode can be described by a set of harmonic oscillators using creation/annihilation operators $\left(c_{j}^{†},c_{i}\right)$ satisfying the commutation relation $\left[c_{i},c_{j}^{†}\right]=\delta_{ij}$, with the Hamiltonian $H_{B}=\sum_{j}\omega_{j}c_{j}^{†}c_{j}$ [28,29,51]. The interaction Hamiltonian between the system and the optical bath is described by

(A1) $$H_{I}=\sum_{j}g_{j}\left(Lc_{j}^{†}+L^{†}c_{j}\right),$$

where L=a is the Lindblad operator representing the optical damping, and $g_{j}$ is the system–bath coupling strength [51]. We have taken the rotating-wave approximation (RWA) for our formalism as we have only considered weak-coupling strengths, a high cavity leakage rate and weakly non-Markovian regimes (for a systematic analysis of the RWA, see, [57,58]).

Making use of the non-Markovian quantum state diffusion (NMQSD) [33,35,51], while assuming that the system is initially uncorrelated with the environment

(A2) $$\partial_{t}|\psi_{t}\left(z^{*}\right)\rangle =\left[-iH_{s}+az_{t}^{*}-a^{†}\bar{O}\left(t,z^{*}\right)\right]|\psi_{t}\left(z^{*}\right)\rangle ,$$

where $O\left(t,s,z^{*}\right)\psi_{t}\equiv \frac{\delta \psi_{t}}{\delta z_{s}}$ and $\bar{O}\left(t,z^{*}\right)\equiv \int_{0}^{t}ds\alpha \left(t,s\right)O\left(t,s,z^{*}\right)$ with the initial condition $O(t,s=t,z^{*})=a$. $\alpha \left(t,s\right)=\frac{\kappa \gamma }{2}e^{-\gamma |t-s|}$ is the Ornstein–Uhlenbeck (O-U) correlation function. $z_{t}^{*}=-i\sum_{j}g_{j}z_{j}^{*}e^{i\omega_{j}t}$ is a colored complex Gaussian process satisfying

(A3) $$M\left[z_{t}^{*}z_{s}\right]=\alpha \left(t,s\right).\;M\left[z_{t}z_{s}\right]=0,$$

where $M\left[\cdot \right]\equiv \int \frac{dz^{2}}{\pi }e^{-{\left|z\right|}^{2}}\left[\cdot \right]$ denotes the ensemble average over the noise $z_{t}$.

To solve (A2), one needs to find the operator $O(t,s,z^{*})$. Under the condition that the memory time is not too long, we expand $O(t,s,z^{*})$ in powers of (t−s) [34]

(A4) $$O\left(t,s,z^{*}\right)=O\left(s,s,z^{*}\right)+\frac{\partial O(t,s,z^{*})}{\partial t}{|}_{t=s}\left(t-s\right)+\cdots ,$$

which is a systematic expansion of the non-Markovian QSD. The zeroth-order term corresponds to the standard Markov QSD when 1/γ→0. We choose the first-order approximation of the operator $O(t,s,z^{*})$ since the memory time is assumed to be short.

At the time point t=s, the expression of the operator $O(t,s,z^{*})$ is given by [34]

(A5) $$\begin{array}{cc} O(s,s,z^{*}) & =a, \end{array}$$

(A6) $$\begin{array}{cc} \frac{\partial O(t,s,z^{*})}{\partial t}{|}_{t=s} & =-i\left[H_{s},a\right]-\int_{0}^{s}\alpha \left(s,u\right)du\left[a^{†},a\right]a, \end{array}$$

where $H_{s}$ is the system Hamiltonian and *a* is the Lindblad operator. At this time point, the operator $\bar{O}$ becomes

(A7) $$\bar{O}=f_{0}\left(t\right)a-f_{1}\left(t\right)i\left[H_{s},a\right]-f_{2}\left(t\right)\left[a^{†},a\right]a,$$

where

(A8) $$\begin{array}{cc} \; & f_{0}\left(t\right)=\int_{0}^{t}\alpha \left(t,s\right)\;ds,\\ \; & f_{1}\left(t\right)=\int_{0}^{t}\alpha \left(t,s\right)\left(t-s\right)\;ds,\\ \; & f_{2}\left(t\right)=\int_{0}^{t}\int_{0}^{s}\alpha \left(t,s\right)\alpha \left(s,u\right)\left(t-s\right)\;du\;ds. \end{array}$$

The explicit form of $f_{i}$ is given by

(A9) $$\begin{array}{cc} f_{0}\left(t\right)\; & =\frac{\kappa }{2}\left(1-e^{-\gamma t}\right), \end{array}$$

(A10) $$\begin{array}{cc} f_{1}\left(t\right)\; & =\frac{\kappa }{2\gamma }\left(1-e^{-\gamma t}-\gamma te^{-\gamma t}\right), \end{array}$$

(A11) $$\begin{array}{cc} f_{2}\left(t\right)\; & =\frac{\kappa^{2}}{4\gamma }\left(1-e^{-\gamma t}-\gamma te^{-\gamma t}-\frac{1}{2}\gamma^{2}t^{2}e^{-\gamma t}\right). \end{array}$$

One can turn (A2) into a master equation by taking the ensemble mean over the noise $z_{t}$ and introducing the reduced density matrix $\rho_{t}=M[|\psi_{t}\left(z^{*}\right)\rangle \langle \psi_{t}\left(z^{*}\right)|]$. When the environment is not far away from Markov, the dependence of the operator $\bar{O}\left(t,z^{*}\right)$ on the noise $z_{t}$ is negligible. Under this approximation, the master equation [34] takes the form

(A12) $$\frac{d}{dt}\rho_{t}=-i\left[H_{s},\rho_{t}\right]+\left[a,\rho_{t}\bar{O}{\left(t\right)}^{†}\right]-\left[a^{†},\bar{O}\left(t\right)\rho_{t}\right].$$

For further details about the derivation of the master equation, see [36,40].

Since the mechanical bath is separate and Markov, it can be added to the master equation by simply including the appropriate Lindblad term, which is given by

(A13) $$\Gamma D\left[b,\rho \right]=\Gamma \{b\rho b^{†}-\frac{1}{2}\left(b^{†}b\rho +\rho b^{†}b\right)\},$$

where $\Gamma /\Omega =10^{-3}$ is the mechanical damping.

Using the first-order approximation of $\bar{O}\left(t\right)$ (A7), the master equation takes the final form

(A14) $$\dot{\rho }=-i\left[H_{s},\rho \right]+\Gamma D\left[b,\rho \right]+\{f_{0}\left(t\right)\left[a\rho ,a^{†}\right]+if_{1}\left(t\right)\left[a^{†},\left[H_{s},a\right]\rho \right]+f_{2}\left(t\right)\left[a^{†},\left[a^{†},a\right]a\rho \right]+H.C.\},$$

where H.C. stands for Hermitian conjugate.
